# Comparative Evaluation of Phenotypic Synergy Tests versus RESIST-4 O.K.N.V. and NG Test Carba 5 Lateral Flow Immunoassays for the Detection and Differentiation of Carbapenemases in *Enterobacterales* and Pseudomonas aeruginosa

**DOI:** 10.1128/spectrum.01080-21

**Published:** 2022-02-02

**Authors:** Diego Josa M, Rafael Leal, Julieth Rojas, Isabel Torres M, Fabián Cortés-Muñoz, German Esparza, Luis Felipe Reyes

**Affiliations:** a Research Group in Cardiovascular Medicine and High Complexity Specialties, Fundación Clínica Shaio, Bogotá, Colombia; b PROASECAL SAS Proficiency Testing Program, Bogotá, Colombia; c Department of Infectious Diseases, Universidad de La Sabana, Chía, Colombia; Hartford Hospital

**Keywords:** carbapenemases, immunoassay, synergy test, boronic acid, EDTA, multidrug resistance

## Abstract

The spread of carbapenem-resistant Pseudomonas aeruginosa and carbapenemase-producing *Enterobacterales* (CPE) has dramatically impacted morbidity and mortality. COVID-19 pandemic has favored the selection of these microorganisms because of the excessive and prolonged use of broad-spectrum antibiotics and the outbreaks related to patient transfer between hospitals and inadequate personal protective equipment. Therefore, early CPE detection is considered essential for their control. We aimed to compare conventional phenotypic synergy tests and two lateral flow immunoassays for detecting carbapenemases in *Enterobacterales* and P. aeruginosa. We analyzed 100 carbapenem-resistant Gram-negative bacilli isolates, 80 *Enterobacterales,* and 20 P. aeruginosa (86 isolates producing KPC, NDM, OXA-48, IMP, and VIM carbapenemases and 14 non-carbapenemase-producing isolates). We performed a modified Hodge test, boronic acid and ethylenediaminetetraacetic acid (EDTA) synergy tests, and two lateral flow immunoassays: RESIST-4 O.K.N.V. (Coris Bioconcept) and NG Test Carba 5 (NG Biotech). In total, 76 KPC, seven VIM, one NDM, one OXA-48, and one isolate coproducing KPC + NDM enzymes were included. The concordance of different methods estimated by the Kappa index was 0.432 (standard error: 0.117), thus showing a high variability with the synergy tests with boronic acid and EDTA and reporting 16 false negatives that were detected by the two immunochromatographic methods. Co-production was only detected using immunoassays. Conventional phenotypic synergy tests with boronic acid and EDTA for detecting carbapenemases are suboptimal, and their routine use should be reconsidered. These tests depend on the degree of enzyme expression and the distance between disks. Lateral flow immunoassay tests are a rapid and cost-effective tool to detect and differentiate carbapenemases, improving clinical outcomes through targeted therapy and promoting infection prevention measures.

**IMPORTANCE** Infections due to multidrug-resistant pathogens are a growing problem worldwide. The production of carbapenemases in Pseudomonas aeruginosa and *Enterobacterales* cause a high impact on the mortality of infected patients. Therefore, it is of great importance to have methods that allow the early detection of these multi-resistant microorganisms, achieving the confirmation of the type of carbapenemase present, with high sensitivity and specificity, with the aim of improving epidemiological control, dissemination, the clinical course to through targeted antibiotic therapy and promoting infection control in hospitals.

## INTRODUCTION

Carbapenem-resistant Gram-negative bacilli, particularly Pseudomonas aeruginosa and carbapenemase-producing *Enterobacterales* (CPE), represent a global public health concern. They are primarily isolated from health care-associated infections (HAIs). The COVID-19 pandemic has favored their spread through prolonged use of broad-spectrum antibiotics, immunomodulatory medications, the massive transfer of patients between institutions, and the inappropriate use of personal protective equipment (1, 2). Carbapenemases are enzymes that can hydrolyze all beta-lactam antibiotics, including carbapenems, to varying degrees. They disseminate using mobile genetic elements (plasmids and transposons). Despite the availability of new carbapenemase inhibitors (e.g., ceftazidime-avibactam), there are limited therapeutic options for metallo beta-lactamases or enzyme co-productions, increasing patient morbidity and mortality (3–5).

Accurate and rapid diagnosis is essential for successful infection prevention strategies and selecting the most appropriate treatment through targeted therapy (6, 7). Recognizing these enzymes specifically influences the therapeutic decision (e.g., combination therapy versus monotherapy), the use of new β-lactamase inhibitors (e.g., Avibactam, relebactam), and the addition of Aztreonam in metallo-beta-lactamases or enzyme co-productions (7–9).

In Colombia and other Latin American countries, different phenotypic tests are used for detecting carbapenemases such as the modified Hodge test (MHT) (10), the carbapenem inactivation method (CIM) (11, 12), and the modified CIM method (mCIM) (13), as well as synergy tests with boronic acid for detecting serine-carbapenemases like KPC and ethylenediaminetetraacetic acid (EDTA) for detecting metallo-beta-lactamases such as NDM or VIM (14). Although these methodologies are inexpensive, they are limited by long turnaround times (18 h to 24 h), false positives caused by membrane permeabilization (e.g., EDTA) or hyperproduction of AmpC (e.g., boronic acid), and false negatives because of adjustment of the distance between disks, low levels of enzyme expression, or co-production of carbapenemases. Moreover, these methodologies cannot discriminate the specific type of enzyme relevant to making a targeted therapeutic decision.

Molecular methods for carbapenemase detection offer rapid and accurate results with high sensitivity and specificity. However, their cost remains a barrier for widespread implementation in clinical laboratories (15). Lateral flow immunoassays, extensively used in the clinical laboratory, have emerged as a cost-effective tool to rapidly detect, characterize, and report the presence of carbapenemases. This method is based on a membrane technology with colloidal gold nanoparticles and monoclonal antibodies directed against an epitope of the five most important carbapenemase families (KPC, VIM, NDM, OXA-48, and IMP), detecting those enzymes with high specificity and sensitivity (16–18).

However, there is little evidence comparing these new detection methods with traditional synergy methods routinely used in clinical practice. This study aims to compare the boronic acid and EDTA phenotypic synergy tests (i.e., conventional processing technics) with two lateral flow immunoassays for detecting carbapenemases in *Enterobacterales* and Pseudomonas aeruginosa.

## RESULTS

A total of 86 carbapenemase-producing Gram-negative isolates and 14 non-producing carbapenem-resistant isolates were tested for detecting and confirming the type of carbapenemases by phenotypic synergy methods and by two brands of lateral flow immunoassays (Table 1).

**TABLE 1:** Distribution of carbapenemase-producing *Enterobacterales* and Pseudomonas aeruginosa isolates used in the study^*a*^

		Type of carbapenemase					Carbapenem-resistant (Non-carbapenemase-producing)
Microorganism	No. (%)	KPC	NDM	OXA-48	VIM	KPC+NDM	
K. pneumoniae	54 (54%)	51				1	2
P. aeruginosa	20 (20%)	2			7		11
E. coli	7 (7%)	6		1			
E. cloacae	6 (6%)	6					
S. marcescens	4 (4%)	4					
K. oxytoca	3 (3%)	3					
K. aerogenes	2(2%)	1					1
*C. koseri*	1 (1%)	1					
C. freundii	1 (1%)	1					
*K. cryocrescens*	1 (1%)	1					
P. mirabilis	1 (1%)		1				
Total	100 (100%)	76	1	1	7	1	14

a KPC, Klebsiella pneumoniae carbapenemase; VIM, verona integron-mediated metallo- β-lactamase; NDM, New Delhi metallo-β-lactamase; OXA, oxacillinase-48-like carbapenemase (OXA-48).

**FIG 1:**
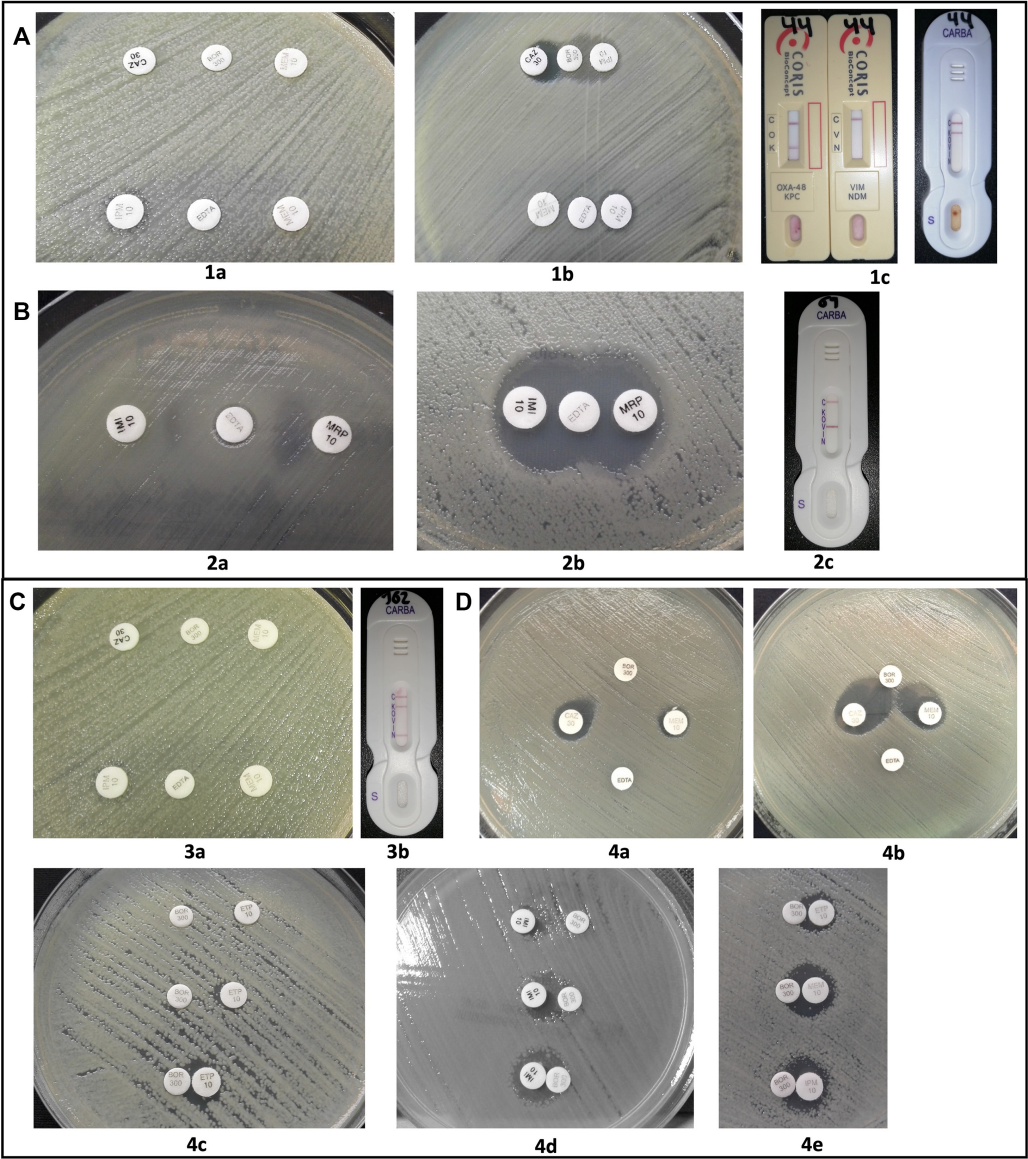
Errors in synergy tests. (A) *KPC-producing* K. pneumoniae not detected with boronic acid and EDTA (1a). Positive result adjusting distance to 5 mm (1b) and positive result by NG Test Carba 5 and *RESIST-4 O.K.N.V* immunoassays (1c). (B) *VIM-producing* P. aeruginosa with inconclusive EDTA result (2a), positive result adjusting distance to 5 mm (2b), confirmation of VIM by immunoassay (2c). (C) K. pneumoniae *with KPC and NDM co-production* without synergistic effect with a boronic acid or EDTA (3a), positivity for both enzymes by immunoassay (3b). (D) *Impact of the distance between disks*: False-negative for serine-carbapenemases with 15 mm (4a) positive result at 10 mm (4b). Differences in boronic acid tests for serin-carbapenemases at 10 mm, 5 mm, and 0 mm distance (4c, 4d). There are differences in synergy between imipenem, meropenem, and ertapenem discs at 0 mm distance (4e).

The concordance of the different methods determined by Kappa index was 0.432 (standard error: 0.117. 95% CI = 0.202 to 0.662), which corresponds to a moderate concordance because of the high variability observed in the results obtained with the phenotypic tests of boronic acid and/or EDTA synergy tests. There were 16% errors corresponding to 16 false negatives for serine-type and metallo-beta-lactamase-type carbapenemases only detected by lateral flow immunoassay (Fig. 1).

### Modified Hodge test and boronic acid and EDTA synergy tests.

Of the 74 KPC carbapenemase-producing *Enterobacterales*, 67 (90.5%) were classified as “serine-type carbapenemases” by boronic acid synergy. From this, 7/74 (9.5%) isolates were falsely negative, and one false-positive result with boronic acid occurred in a non-carbapenemase-producing carbapenem-resistant Klebsiella pneumoniae isolate (Table 2). MHT was positive in 69/74 (93.2%) of *Enterobacterales*, and false-negative results were obtained in five isolates (6.8%) even when only KPC was detected.

**TABLE 2:** Results of synergy methods and immunoassay tests for *Enterobacterales* and Pseudomonas aeruginosa^*a*^

*Enterobacterales n = 80*		Immunoassay				Phenotypic synergy tests					
		RESIST-4 O.K.N.V.		NG Test Carba 5		MHT		APB		EDTA	
Carbapenemases	n	Pos	Neg	Pos	Neg	Pos	Neg	Pos	Neg	Pos	Neg
KPC	74	74	0	74	0	69	5	67	7	0	74
NDM	1	1	0	1	0	1	0	0	0	1	0
VIM	0										
OXA-48	1	1	0	1	0	1	0	0	1	0	1
KPC + NDM co-production	1	1	0	1	0	1	0	0	1	0	1
Total	77	77	0	77	0	72	5	67	9	1	76
Non-carbapenemase-producing	3	0	3	0	3	1	2	1	2	0	3
Total	80										

Pseudomonas aeruginosa *n = 20*		Immunoassay				Phenotypic tests	
		RESIST-4 O.K.N.V.		NG Test Carba 5		EDTA	
Carbapenemases	n	Pos	Neg	Pos	Neg	Pos	Neg
KPC	2	2	0	2	0	0	2
NDM	0						
VIM	7	7	0	7	0	2	5
Total	9	9	0	9	0	2	7
Non-carbapenemase-producing	11	0	11	0	11	2	9
Total	20						

a MHT, Modified Hodge test; APB, boronic acid synergy; EDTA, EDTA synergy; Pos, positive; Neg, negative; KPC, Klebsiella pneumoniae carbapenemase; VIM, (verona integron-mediated metallo- β-lactamase); NDM, New Delhi metallo-β-lactamase; OXA, oxacillinase-48-like carbapenemase (OXA-48).

For P. aeruginosa, the EDTA synergy test showed conflicting results. A total of 5/7 (71.4%) of VIM-producing isolates were falsely negative with the EDTA test (Table 2). In non-carbapenemase-producing carbapenem-resistant P. aeruginosa, 2/11 (18.2%) isolates were false positive with EDTA. The OXA-48 producing E. coli was negative for boronic acid and EDTA synergy tests.

The K. pneumoniae isolate co-producing KPC + NDM enzymes presented falsely negative results with the two inhibitors (boronic acid and EDTA) (Fig. 1, see 3a, 3b).

In our study, the phenotypic boronic acid synergy tests yielded a sensitivity of 84.5% and a specificity of 80.2% (PPV: 80,5%, NPV: 76,3%), and the EDTA tests yielded a sensitivity and specificity of 81% and 78.1%, respectively (PPV: 79,3%, NPV: 79.9%).

### Lateral flow immunoassay tests.

Both immunoassays evaluated were able to correctly detect and classify the different types of carbapenemases in *Enterobacterales* and P. aeruginosa. There were no differences in detection of KPC, VIM, NDM, and OXA-48 between RESIST-4 O.K.N.V and NG Test Carba 5. The sensitivity and specificity of the immunoassay tests was 100% (PPV: 100%, NPV: 100%) (Table 2).

All carbapenemase-producing isolates, KPC, NDM, VIM, and OXA-48, yielded a rapid result with a purple or red band on the nitrocellulose membrane of the cassette of each of the brands evaluated. Only additional time was required for reading (total time: 20 min) a faint color band for type VIM carbapenemases in the RESIST-4 O.K.N.V immunoassay.

All 14 carbapenem-resistant isolates by mechanisms other than carbapenemase production were negative for immunoassays, unlike synergy methods. The latter yielded 3/14 false-positive results (one false positive with boronic acid and two false positives with EDTA) (Table 2).

### Disk diffusion synergy tests distance adjustments.

For the 16 carbapenemase-producing isolates not detected by synergy methods, repeated synergy tests were performed with boronic acid and EDTA by placing the discs at distances of 10 mm, 5 mm, and 0 mm from the carbapenemic disk. This strategy allowed the detection of carbapenemases in nine isolates: two were positive with boronic acid at 10 mm, one with EDTA at 10 mm, and six were positive only when the discs were placed at a distance of 0 mm with a very slight synergistic effect toward the boronic disc (Fig. 1, see 4a to 4d). The remaining seven isolates showed no change at any distance. In the boronic acid assays with different carbapenems disks at 0 mm distance, a stronger synergistic effect could be observed with the meropenem disks (Fig. 1, see 4e).

## DISCUSSION

Carbapenemase production is one of the most successful resistance mechanisms in Gram-negative bacteria. Rada et al. describe the molecular distribution and characterization of beta-lactamases in Colombia from 2001 to 2016, reporting that KPC is the most frequent one, followed by NDM-1 metallo-beta-lactamase (19). These data are consistent with the results of our study, where the most frequent carbapenemase reported in *Enterobacterales* isolates was KPC in 76% of the isolates. In P. aeruginosa, the most frequent enzyme was VIM, with 7% of the isolates.

The accurate detection of carbapenemases is a challenge for clinical microbiology laboratories in Colombia, which mainly use phenotypic assays for capture (THM and mCIM or carba NP) and differentiation based on inhibitors with boronic acid and EDTA (13, 20). Recently, lateral flow immunoassay has emerged as a cost-effective alternative to detect and differentiate the most important carbapenemase families (16, 18). The tests used in this study were RESIST-4 O.K.N.V (Coris Bioconcept Belgium), which detects four carbapenemase types (OXA-48, KPC, VIM, NDM) and NG Test Carba 5 (NG Biotech, Guipry, France), which detects five carbapenemase types (OXA-48, IMP, KPC, VIM, NDM) in *Enterobacterales* and P. aeruginosa directly from colonies on agar (16, 18).

There was a moderate concordance between phenotypic synergy methods versus PCR in this study because of false positives and negatives in some isolates. The sensitivity and specificity obtained in the boronic acid synergy test were similar to that reported in other comparative studies by Tamma et al., approximately 85% to 88% (21). Sensitivity and specificity have been reported for the EDTA test 51% and 95%, respectively (22).

Synergy tests are highly dependent on the distance the disks are placed. As was evident in the 16 false-negative cases, when placing inhibitors closer to carbapenems as an alternative restored the positivity of the test in most cases; however, in some isolates, it was only possible to see synergy effect if disks were placed entirely close for each other; evidencing the low sensitivity of these synergy tests. Unfortunately, it is challenging to standardize distance because it will depend on the degree of enzyme hydrolysis (21, 22).

Another essential issue of synergy methods lies in the limitation to use boronic acid to detect class A carbapenemases in glucose non-fermenting Gram-negative bacilli (e.g., P. aeruginosa) because it may provide false-positive results for hyperproduction of AmpC, reducing the ability to differentiate between serine carbapenemases and AmpC production (22). On top of that, OXA-48 enzymes pose a more significant challenge as no specific inhibitors favor their silent dissemination.

There are microbiology labs that only perform boronic acid and EDTA. According to the issues described above, we recommend performing capture tests when precision methods such as PCR or immunoassay are not available.

Co-production of carbapenemases constitutes a significant diagnostic and therapeutic challenge. Synergy-based boronic acid and EDTA tests used in Colombia and suggested in guidelines published by the National Institute of Health (INS) (23) cannot detect co-productions because of its variable expression (Fig. 1 3a-3b). This limitation was previously reported in one isolate coproducing NDM and KPC, as EDTA could detect only the metallo-beta-lactamase. Still, serine-type KPC carbapenemase was not detected by boronic acid (24). According to the present work results, boronic acid synergy tests can provide false negatives even in isolates producing only one type of enzyme (KPC) as confirmed by PCR. Co-productions are only detectable with high-precision methods like PCR and lateral flow immunoassay, which indicated positivity for both KPC and NDM enzymes in one isolate of K. pneumoniae (24).

The clinical and epidemiological impact of false-negative results for carbapenemases needs to be emphasized. Evidence shows favorable clinical outcomes in Gram-negative infections caused by isolates coproducing class A and B enzymes using ceftazidime-avibactam + aztreonam combinations that could only be justified using precision medicine (25, 26).

The advantages of lateral flow immunoassay include a rapid turnaround time (15 min from assembly), ease of processing and interpretation, and greater sensitivity and specificity reported in various studies compared with PCR (95% to 100%) (16–18), which in our work correlated 100% with molecular characterization by PCR. Moreover, they allow the detection of specific types of enzymes that, along with appropriate antimicrobial susceptibility testing, favor targeted therapy with new inhibitors such as ceftazidime-avibactam and its combination with aztreonam in isolates producing metalloenzymes resistant to aztreonam or isolates coproducing class A+B carbapenemases (26).

As a limitation, this study was conducted in a single-center with a small number of isolates coproducing enzymes and small numbers of P. aeruginosa producing KPC, and the variety of carbapenemases different from KPC, even the number of non-carbapenemase-producing isolates. At the time of the study, we took the strains that we conserved in the laboratory, both carbapenemase-producing and non-producing; no IMP was identified in the study, so the ability of NG Test Carba 5 to detect IMP could not be evaluated. Most are KPC-type carbapenemases because our country is endemic for KPC. However, this is the first study carried out in a reference hospital in Colombia. “The clonal relationship between isolates has not been determined. New effective phenotypic methods such as mCIM and eCIM; which we did not perform in our study, could be used and incorporated for the detection and confirmation of carbapenemases; thus, further studies including these methods are warranted.”

In conclusion, we highlight the suboptimal performance of boronic acid and/or EDTA synergy tests to capture and differentiate carbapenemases. We recommend that routine use in microbiology laboratories be reconsidered, especially in critically ill patients. Lateral flow immunoassay tests are a rapid and cost-effective tool for characterizing carbapenemases where precision medicine has proven to be the new standard of care to improve clinical outcomes and contain their spread in hospital settings.

## MATERIALS AND METHODS

### Bacterial isolates.

One-hundred non-repetitive and consecutive isolates of carbapenem-resistant Gram-negative bacilli collected from different sample types between 2015 and 2019 were selected from the culture collection of the microbiology laboratory the Fundación Clínica Shaio, Bogotá, Colombia. Eighty isolates of *Enterobacterales* and 20 of P. aeruginosa were included. All isolates were characterized for carbapenemase production by PCR (reference method for comparison) against KPC, NDM, OXA-48-like, IMP, and VIM. Of the 80 *Enterobacterales* isolates, 77 were carbapenemase producers: (KPC = 74, NDM =1, OXA-48 = 1, and one isolate co-producing KPC + NDM) and three non-carbapenemase-producing carbapenem-resistant *Enterobacterales* (non-CPE) were selected. Of the P. aeruginosa isolates, nine carbapenemase-producing isolates (VIM = 7, KPC = 2) and 11 non-carbapenemase-producing carbapenem-resistant isolates were included for the study (Table 1). The study period was from November 2019 to March 2020.

### Bacterial isolates recovery.

The isolates included in the study were retrieved using a slow thawing process, with subculture recovery of an aliquot (1 mL) of the strain in a tube containing 3 mL of thioglycolate broth and incubated at 37°C for 24 h. Subsequently, the suspension was plated on MacConkey agar and incubated at 37°C for 18 h to 24 h.

### Phenotypic tests and immunoassays performed.

**(i) Modified Hodge Test (MHT).** Using the indicator strain E. coli ATCC 25922, a 0.5 McFarland suspension was prepared in saline and diluted 1:10. Then a Mueller-Hinton agar plate was inoculated for the routine disk diffusion procedure. The plated dried for 3 to 10 min, and a 10 μg disk of meropenem was placed in the center. Using a 10-μL loop, three to five colonies of each culture of *Enterobacterales* were picked to inoculate a straight line out from the edge of the disk. Plates were incubated at 35 ± 2°C in ambient air for 16 h to 20 h.

**(ii) Boronic acid sensi-discs synergy test (Britania).** For *Enterobacterales*, a 0.5 McFarland suspension was prepared and inoculated on Mueller–Hinton agar. Then, disks of boronic acid 300 µg meropenem (10 µg) and ceftazidime (30 µg) were placed, leaving 15 mm edge-to-edge distance following the recommendations of the Colombian National Institute of Health (INS, for their Spanish acronym). Plates were incubated at 35 ± 2°C in ambient air for 16 h to 20 h.

**(iii) Ethylenediaminetetraacetic acid (EDTA) discs synergy test (Britania).** For *Enterobacterales and* P. aeruginosa, a 0.5 McFarland suspension was prepared and inoculated on Mueller–Hinton agar. Then, disks of EDTA (372 µg), meropenem (10 µg), and imipenem (10 µg) were placed, leaving 15 mm edge-to-edge distance following the Colombian INS recommendations. Plates were incubated at 35 ± 2°C in ambient air for 16 h to 20 h.

**(iv) RESIST-4 – O.K.N.V lateral flow immunoassays (Coris BioConcept, Gembloux, Belgium).** For *Enterobacterales* and P. aeruginosa isolates, 12 drops of the buffer solution were added to a plastic tube. Then, using a loop, three colonies of each isolate were emulsified, and the preparation was shaken for homogenization. Three drops of this suspension were dispensed into the sample well of the cassette, and the test was left to run for a maximum of 15 min. Reading was performed according to the manufacturer’s package insert.

**(v) NG-Test CARBA 5 lateral flow immunoassay (NG Biotech, Guipry, France).** For *Enterobacterales* and P. aeruginosa isolates, five drops of the buffer solution contained in the kit were added to a plastic tube. Subsequently, three colonies were emulsified with a loop, and the preparation was shaken for homogenization. Then, with a pipette, 100 μL of this suspension was dispensed into the cassette sample well. The test was left to run for a maximum of 15 min and read as per the manufacturer’s package insert.

### Disk diffusion synergy distance adjustments for isolates with discordant results (false negatives).

For carbapenemase-producing isolates that presented negative results by boronic acid and/or EDTA synergy tests, a modification of the method was performed, placing boronic acid (300 µg) and meropenem (10 µg) disks closer at different distances (10 mm, 5 mm, and 0 mm edge-to-edge). In the case of EDTA synergy, assays were performed with disks of EDTA (372 µg), meropenem (10 µg), and imipenem (10 µg) closer at different distances (10 mm, 5 mm, and 0 mm edge-to-edge).

**Quality control.** Klebsiella pneumoniae ATCC BAA 1705 (_KPC-2_), K. pneumoniae ATCC BAA 2146 (_NDM-1_), K. pneumoniae ATCC BAA1706, E. coli ATCC 25922, and P. aeruginosa ATCC 27853 were used as controls.

All tests were performed consecutively by the same microbiology laboratory personnel.

### Statistical analysis.

Concordance between the two tests was assessed by calculating the Kappa coefficient (κ), with its corresponding 95% confidence interval. Statistical analyses were performed using STATA v. 15 software (StataCorp, College Station, TX).
